# Feasibility of real-time cine cardiac magnetic resonance imaging to predict the presence of significant retrosternal adhesions prior to redo-sternotomy

**DOI:** 10.1186/s12968-019-0576-x

**Published:** 2019-10-31

**Authors:** Riad Abou Zahr, Vasu Gooty, Animesh Tandon, Gerald Greil, Timothy Pirolli, Ryan Davies, Robert Jaquiss, Claudio Ramaciotti, Tarique Hussain

**Affiliations:** 1 Department of Pediatrics, University of Texas Southwestern Medical Center, Dallas, USA; 2 Department of Surgery, University of Texas Southwestern Medical Center, Dallas, USA

**Keywords:** Retrosternal adhesions, Real-time cine, Redo sternotomy

## Abstract

**Background:**

Injury to vital structures posterior to the sternum is a complication associated with redo sternotomy in congenital cardiac surgery. The goal of our study was a novel evaluation of real-time cine cardiovascular magnetic resonance (CMR) to predict the presence of significant retrosternal adhesions of cardiac and vascular structures prior to redo sternotomy in patients with congenital heart disease.

**Methods:**

Twenty-three patients who had prior congenital heart surgery via median sternotomy had comprehensive CMR studies prior to redo sternotomy. The real time cine (RTC) sequence that was used is an ungated balanced steady-state free precession (bSSFP) sequence using SENSitivity Encoding for acceleration with real-time reconstruction. Spontaneously breathing patients were instructed to take deep breaths during the acquisition whilst increased tidal volumes were delivered to mechanically ventilated patients. All patients underwent redo cardiac surgery subsequently and the presence and severity of retrosternal adhesions were noted at the time of the redo sternotomies.

**Results:**

Median age at the time of CMR and operation were 5.5 years (range, 0.2–18.4y) and 6.1 years (range, 0.3–18.8y) respectively. There were 15 males and 8 females in the study group. Preoperative retrosternal adhesions were identified on RTC in 13 patients and confirmed in 11 (85%) at the time of surgery. In only 2 patients, no adhesions were identified on CMR but were found to have significant retrosternal adhesions at surgery; false positive rate 15% (CI 0.4–29.6%), false negative rate 20% (CI 3.7–36.4%). The total classification error of the real time cine sequence was 17% (CI 1.7–32.4%) with an overall accuracy of 83% (CI 67.7–98.4%). Standard breath-hold cine images correlated poorly with surgical findings and did not increase the diagnostic yield.

**Conclusions:**

RTC imaging can predict the presence of significant retrosternal adhesions and thus help in risk assessment prior to redo sternotomy. These findings complement the surgical planning and potentially reduce surgical complications .

## Background

Sternal re-entry injury to cardiovascular structures is a well-known complication associated with repeat sternotomy in cardiac surgery [1]. This is usually due to extensive adhesions caused by prior surgeries. The risk of injury to cardiac and vascular structures is around 1–5% in different series [2–5]. Presence of a conduit as well as the number of previous sternotomies are significant risk factors for reentry-associated injury [2]. Thus assessment of retrosternal adhesions prior to redo surgery provides valuable information that can guide surgical planning to avoid unexpected complications.

The use of computed tomography (CT) in congenital heart disease has been demonstrated to be a valuable tool for surgical planning of repeat sternotomies and to reduce complications at the time of re-entry. In one study, cardiovascular magnetic resonance (CMR) was found to be inferior to CT in determining retrosternal adhesions [6]. Historically, CMR was considered non-diagnostic of retrosternal adhesions due to sternal wire artifact [7]. With advancement in technology, CMR became capable of predicting the severity of retrosternal adhesions using tagged cine sequences [8]. Due to the fact that direct visualization of retrosternal adhesions may not be possible at all times, tethering or deformation of mediastinal structures on cine CMR or CT imaging has been used as a surrogate instead [9]. However, transverse imaging of the heart independent of chest wall movements can miss significant adhesions. We hypothesized that mid-sagittal dynamic imaging of cardiac and chest wall movements would enable identification of significant retrosternal adhesions. The goal of this study is to demonstrate the feasibility of real-time cine (RTC) CMR during deep breathing for visualization of retrosternal adhesions. Furthermore, RTC imaging was compared to standard breath-hold cine CMR imaging.

## Methods

We conducted a retrospective study at Children’s Medical Center and the University of Texas Southwestern Medical Center, Dallas, Texas from November 2017 to February 2019. Clinically indicated comprehensive CMR studies were performed for patients with prior congenital heart surgery on a 1.5 T scanner (Philips Healthcare, Best, The Netherlands) using a 32-channel torso coil as part of a pre-surgical evaluation. Included in the standard CMR protocol were breath-hold (BH) cine imaging of the ventricular short axis (SAx) and the right ventricular outflow tract (RVOT) in a sagittal-oblique orientation. In addition to the standard CMR sequences, a RTC sequence was used. All patients underwent redo sternotomies for indicated cardiac surgeries subsequently and the presence of significant retrosternal adhesions were noted. The study was approved by the local ethics committee (IRB STU 032016–009) for retrospective analysis of CMR and clinical data.

### Real time cine sequence

The RTC sequence that was used is a two-dimensional dynamic scan consisting of balanced steady state free precession (bSSFP) with a flip angle of 50 degrees, repetition time of 2.2 ms, echo time of 1.1 ms, SENSE acceleration factor of 2.5, without cardiac synchronization or respiratory compensation, acquiring 150 dynamic scans over 12–18 s (i.e. true temporal resolution 80–120 ms) in a 10–20 mm thick single slice across the midline sagittal plane, voxel size 2.5 × 2.5 and signal to noise ratio of 1. Cartesian sampling was used to fill K-space. A sagittal orientation with readout along anterior-posterior direction was chosen. Spontaneously breathing patients were instructed to take deep breaths during the acquisition while increased tidal volumes were delivered to mechanically ventilated patients.

### Identification of significant adhesions by CMR

**Fig. 1:**
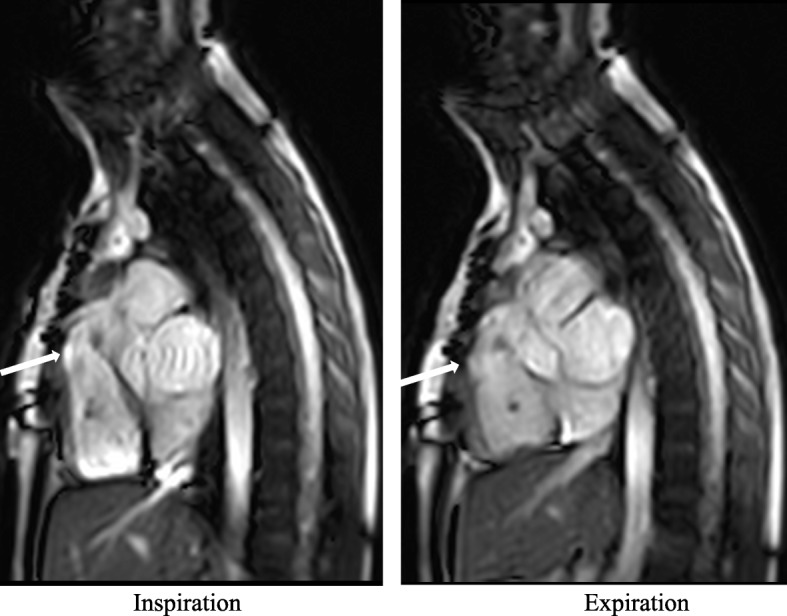
Real-time cine images of a patient with previous tetralogy of Fallot repair with transannular patch. The arrow points to the attachment of the RVOT to the posterior aspect of the sternum. On the left, during deep inspiration, the sternum and RVOT maintain the same position as on expiration (right). Furthermore, during inspiration the RVOT appears deformed and elongated. This suggests that the RVOT is adhered to the sternum by dense adhesions, limiting independent movement


Significant adhesions were detected on the RTC sequence by the movement of retrosternal cardiac or vascular structures in concert with the sternum during deep breathing. During inspiration the diaphragm descends; whereas the anterior chest wall, which includes the sternum, is displaced in the cranial, lateral and, most importantly, ventral directions. In normal subjects, this movement allows for descent of the heart, sliding freely beneath and independent of the sternum, as the diaphragm flattens. In patients with significant cardiac/vascular adhesions to the sternum, the normal heart displacement will be limited and specific cardiac structures, usually the right ventricle (RV), RVOT or an RV-pulmonary artery conduit, will be tethered to the sternum with limited independent motion. These retrosternal structures will appear deformed and elongated during inspiration (Fig. 1; Additional file 1: Video S1 & Additional file 2: Video S2). Two observers (RA and VG) reviewed all the images independently at two different time points for presence or absence of significant adhesions. Intra- and inter-observer reliability were assessed using the Cohen’s Kappa (κ) statistic. Adhesions were identified on the standard BH cine sequences by two independent observers (VG and TH) in a blinded fashion without reviewing the surgical or RTC data. Descriptive statistics were used to present the data. Medians and interquartile ranges (IQR) were used to describe continuous variables. Sensitivity, specificity, false positive and negative rates were calculated.

**Additional file 1: Video S1.** RTC CMR demonstrates significant retrosternal adhesions where the RVOT is tethered to the sternum and during inspiration becomes deformed and elongated.



**Additional file 2: Video S2.** RTC CMR demonstrates no significant retrosternal adhesions.


## Results

Twenty-three patients with prior congenital heart surgery via median sternotomy underwent CMR and subsequently surgery (Table 1). Median ages at the time of CMR and surgery were 5.5 years (range, 0.2–18.4y, IQR 5.4y) and 6.1 years (range, 0.3–18.8y, IQR 5.5y) respectively. There were 15 males. The patients’ primary diagnoses included: tetralogy of Fallot, hypoplastic left heart syndrome and atrioventricular septal defect. Surgical procedures included pulmonary valve replacement, Fontan palliation, mitral valve repair, RV to pulmonary artery homograft, Glenn palliation.

**Table 1:** Demographic Data

Patient Characteristics	Results
Total number of patients	23
Gender (male/female)	15/8
Median age at CMR (years)	5.5
Median age at redo surgery (years)	6.1
Primary Diagnoses	
TOF	15
HLHS	5
AVSD	1
DORV	1
SV	1
Planned Redo Surgery	
PVR	9
RV-PA homograft replacement	6
Fontan	5
MVR	1
Glenn	1
TV repair	1

*Abbreviations*: *TOF* tetralogy of Fallot, *HLHS* hypoplastic left heart syndrome, *AVSD* atrioventricular septal defect, *SV* functional single ventricle, *PVR* pulmonary valve replacement, *RV-PA* right ventricle to pulmonary artery, *MVR* mitral valve repair, *TV* tricuspid valve

Preoperative retrosternal adhesions were identified on RTC images in 13 (57%) patients and subsequently confirmed in 11 (85%) at surgery. The false positive rate was 15% (CI 0.4–29.6%). CMR demonstrated lack of significant adhesions in 10 patients and surgery confirmed 8 (80%) of them. The 2 remaining patients had significant retrosternal adhesions at surgery. The false negative rate was 20% (CI 3.7–36.4%). The total classification error of the real time cine sequence was 17% (CI 1.7–32.4%) with an overall accuracy of 83% (CI 67.7–98.4%). The sensitivity of detecting retrosternal adhesions using the RTC sequence was 85% (CI 70.4–99.6%) and specificity was 80% (CI 63.7–96.4%) (Table 2). Five patients had injury to cardiovascular structures during their redo surgeries, 4 of which were at the time of sternotomy and 1 after entering the thoracic cavity, not related to sternal adhesions. Three (75%) of the 4 with injury to cardiovascular structures at the time of sternotomy had evidence of significant adhesions identified on CMR. There was substantial or excellent intra-observer agreement (κ = 0.7 & 0.8) and excellent inter-observer agreement (κ = 0.8) between reviewers.

**Table 2:** Retrosternal adhesions identified by RTC vs Surgery

	Surgery Adhesions	Surgery No Adhesions	Total
RTC: Adhesions	11	2	13
RTC: No Adhesions	2	8	10
Total	13	10	23

*RTC* real time cine

Upon review of the BH cine images for adhesions, 2 studies were considered indeterminate. There was poor correlation between BH cine and surgical findings with a sensitivity of 45% (CI 24–66%), specificity of 40% (CI 19–61%), false positive rate of 54% (CI 33–75%), and a false negative rate of 60% (CI 39–81%) (Table 3). BH cine had no advantage over RTC in any of the patients. Furthermore, combining RTC with BH cine images did not improve the correlation with surgery when compared with RTC alone.

**Table 3:** Retrosternal adhesions identified by BH cine vs Surgery

	Surgery Adhesions	Surgery No Adhesions	Total
BH Cine: Adhesions	5	6	11
BH Cine: No Adhesions	6	4	10
Total	11	10	21

*BH* breath hold cine included short axis and right ventricular outflow tract orientations

## Discussion

In this study we demonstrate the utility of ungated RTC CMR imaging to identify significant retrosternal adhesions in patients with previous sternotomies for congenital heart disease. Choosing a midline sagittal plane orientation allowed for a relatively short scan time. Imaging during deep breathing yielded superior results when compared to regular breath-holding. Our results agreed well with intraoperative findings and CMR was able to predict the presence or absence of adhesions in ≥80%. Thus, this imaging technique is a valuable tool in pre-operative planning and can guide surgeons to take precautionary measures such as cannulation for cardiopulmonary bypass via femoral vessels before performing the redo sternotomy.

This is the first published report to use RTC CMR to evaluate retrosternal adhesions during both cardiac and breathing motions. CT and CMR have been used to evaluate retrosternal adhesions via different techniques. Pre-operative CT has been shown to reduce the rate of surgical complications [10]. Classically, the distance between the sternum and the structure immediately posterior to it was identified as a marker of adhesions [6]. Furthermore, presence of retrosternal fat on static CT is considered a sign of less significant adhesions, while absence of fat suggests severe adhesions [7, 11, 12]. Malguira et al identified adhesions as linear fibrous bands on static retrospective electrocardiogram (ECG)-gated CT [9]. Adhesions were confirmed by detecting deformation and tethering of mediastinal structures on cine CT imaging. Dynamic 4D CT imaging is a relatively new technique that assesses differential mobility to identify tethering between sternum and underlying structures during breathing [13]. Image acquisition occurs while patient is spontaneously breathing for two respiratory cycles over few seconds. Using this modality, Narayanan et al. demonstrated excellent correlation with intraoperative findings without false negative cases [13]. Similar to the CMR RTC technique, the 4D CT incorporates cardiac and respiratory motion to determine tethering; however, it involves a significantly higher radiation dose than coronary CT angiogram [13]. CMR has been used to detect and grade the severity of retrosternal adhesions using tagged cine imaging with aid of a finite element model [8]. Yoshioka et al were able to calculate the strain at the surface of the RV on the basis of tag displacement over the cardiac cycle with acceptable correlation at the time of surgery [8]. This method utilized special software that may not be widely available.

RTC CMR has been successfully used in several clinical scenarios including cardiac arrhythmias, impaired breath holding ability or poor ECG gating [14]. RTC allows ventricular functional assessment comparable to bSSFP [15–18]. The utility of RTC lies beyond assessment of intracardiac anatomy, it has been widely used in thoracic imaging of extra-cardiac chest tumors [19–21]. Lee et al have determined that the combined use of non-breath hold cine and static CMR for assessing mediastinal and chest wall tumor invasion have higher specificity and better diagnostic confidence than that of a static CMR alone [21].

We did not attempt to grade the severity of adhesions due to limitations of spatial resolution. Another limitation of this study is the small sample size. Further improvement to this sequence that yields higher image quality, a larger sample size and a prospective design may allow for detailed grading of adhesions and more generalizability.

## Conclusion

The feasibility of deep breathing mid-sagittal RTC CMR for preoperative detection of significant retrosternal adhesions among patients with prior congenital heart surgery has been demonstrated. This sequence is readily available on all scanners and does not require advanced software. The ability to detect significant adhesions on routine CMR gives additional incremental clinical information. This information allows improved prognostication, preparation and choice of appropriate intervention/ bypass strategy.

## Data Availability

The data that supports the findings of this study is available from the senior author TH upon request.

## Abbreviations

4D: 4 dimensional
BH: Breath-hold
bSSFP: Balanced steady state free precession
CI: Confidence interval
CMR: Cardiovascular magnetic resonance
CT: Computed tomography
ECG: Electrocardiogram
IQR: Interquartile range
IRB: Institutional review board
RTC: Real-time cine
RV: Right ventricle/right ventricular
RVOT: Right ventricular outflow tract
SAx: Short axis
